# Pressure Anesthesia

**Published:** 1904-04-15

**Authors:** R. B. Tuller

**Affiliations:** Chicago, Ills.


### PRESSURE ANESTHESIA.

BY R. B. TULLER., D.D.S., CHICAGO, ILLS.

Pressure anesthesia is a term applied to a process of forcing a fluid medicament into tooth structure that will produce an anesthetic or obtunded condition. Since some claim that this anesthetic or benumbed condition can be produced by the use of pure water, one might conclude, if that be true, that the benumbing was produced by the pressure per se, and not by any particular virtue in the liquid. The term pressure anesthesia, in that case, might be correct; and possibly it may be correct in any event, since the condition of anesthesia is due to the pressure as well as the influence of the drug, as in the forcing in of a cocain solution. I have never attempted to produce anesthetic results with water alone, because I reason that if water will produce such results, we may more certainly insure results by the introduction of cocain with the water.

I am inclined to use the term *pressure cataphoresis* and have used it to some extent. Cataphoresis is an infiltration of the tissues from the external, and is cataphoresis, I take it, whether produced by the electric current or by pressure. We are apt to associate cataphoresis with electricity simply because our familiar acquaintance with the term came with its introduction into dentistry with the electric current.

I wish to discuss the results produced by forcing certain medicaments or drugs in liquid form, such as cocain solution, into tooth tissue; or, to be more exact, into and through the dentine and into the pulp.

That this method has merit I think no one can doubt. That it is not more widely practiced I think is due to a great majority of dentists not understanding either the technique of the operation or the theory of its action. Many can not understand, and hesitate to believe, that a liquid substance can be forced into the hard dentine of a tooth, and even through it into the pulp. But that it can be done has been proved over and over by many, who are now using the method frequently to free their patients of the pain incident to excavating and filling sensitive teeth and the removal of exposed pulps. Other "doubting Thomases" will unhesitatingly declare that no one can press on an exposed live pulp without producing pain, and when I state openly that I have done it again and again without one twinge of pain, I expect to hear doubts expressed.

My first attempts at pressure anesthesia were failures; and my inclination was to pronounce it a fake. At length, in a favorable case, I very unexpectedly succeeded, and so perfectly that I was charmed, as was also my patient. Then I tried to reason out why I had failed so many times, and at the same time I perhaps got a hint that my medicament must be most securely confined under the pressure; and, gentlemen, that is the whole secret. Some of you know that, and I presume some do not, or at least do not comprehend. I found many cavities which had to be patched up with cement or modeling compound or some such thing, to make a pocket or well with walls that would not leak. This often took a lot of time, and when at length pressure was applied with soft rubber over the saturated cotton in the cavity, and no desired results reached and time again wasted, I often found the hour allotted to the sitting was up, or so nearly so that I could not do what I had intended, and I had to send my patient away rather disgusted and lacking faith in me, as I then lacked faith in the painless process I had attempted.

Still I met with success enough in some cases to feel that the method was all right if I could make more of the conditions good. I have not made them all good by any means, by my method, but I have made many of them good.

I will say that in a reasonably deep, cup-shaped cavity, with good, unfissured walls, you need not seek anything better than the plug of soft rubber behind your saturated cotton, if the plug is made to fit the opening and a suitably shaped and large enough instrument is used so that the liquid, secure from leaking otherwise, does not regurgitate. In such a cavity with certainty of confinement, I do not think any one should fail, if he persists long enough in the effort and exert a force strong enough.

But we find, as I have said, many cavities so shaped that we can not confine liquid in them any more than you can on the top of a ten-cent piece. With many such cavities to contend with, the thought came to me to try and confine my solution without regard to faulty walls or any kind of tooth walls. I fashioned a cup in a soft piece of rubber, and fastening this to an instrument and putting my cocain solution in the cup, and applying it, met with very quick and satisfying results. This was in a case of pulp exposure. The little rubber cup was placed over the exposure very gently and held a moment before beginning pressure. The exposed surface of a pulp will take up a little cocain and become so benumbed that it will tolerate a little pressure, which may be gradually increased until one may be pressing with a force of many pounds. This should be kept constant for a little while; and to make assurance doubly sure, I repeat the dose of cocain and send it home. If all conditions are good, the pulp may be removed painlessly in five minutes. If conditions are faulty it may take longer. I have failed for a matter of ten or fifteen minutes in some cases, and even longer, and then came out successful. So success depends, to some extent, upon persistency, as the fault that requires it can not always be determined and corrected.

Sometimes a rubber tip may split and leak. They should be carefully and frequently inspected and fresh ones substituted.

Now I labored under a delusion that I believe many others labor under, and that is that in obtunding sensitive dentine the obtunding agent must be in contact with the walls of the entire cavity or one could not hope for results; but on a hint from Dr. G. V. Black, I proved to myself that such is not the case, for on placing my little cup of cocain solution on the bottom of a broad cavity immediately over the pulp chamber, I soon got obtunded results, not only just where I pressed, but in the entire cavity; and this led me to the conclusion that my solution passed through the dental tubuli to the pulp, which becoming influenced, influenced in the same way all the nerve fibrils or prolongations of odontoblasts that radiate from it, so that excavating in any part of the tooth was a painless operation.

Of course it is apparent to every one that when such a condition is brought about in a tooth, the greatest care must be taken not to uncover the pulp accidentally or injure it by the too persistent friction of rapidly-revolving burs. If an operator does not feel safe in his ability not to uncover a benumbed pulp, he had better not anesthetize it, and in fact, as I have said, I do not advocate its use in a multitude of cases where we can do very well without it.

But there are again many cases where it is a boon both to the dentist and his patients. I am satisfied that no pulp is injured by the slight influence necessary to produce thoroughly obtunded results. Neither do I believe that with the minute quantity used we are in much danger of toxic effects through cocain in the circulatory system. I have never heard of any trouble of the kind, not even when we infiltrate a pulp to the apex for immediate extirpation. We might say we take the pulp out too quickly, but to put another phase on that, I will say that I have anesthetized an exposed and aching pulp, and then not having time to do more have packed the cavity with gutta-percha hard pressed on the exposure, leaving it for a more convenient time (twenty-four to forty-eight hours) to extirpate and have had no occurrence of pain or unpleasant results. I am not prepared to say that this is good practice, but several cases have turned out well—no pain in the interval of waiting and none in removal of pulp when it suited my convenience to do it, except one case that was a triffle sensitive near the apex.

A few weeks ago I had occasion to remove the pulps of some eight or nine teeth for bridge supports, the case being one of seriousl mal-occlusion that could only be regulated by cutting off sound teeth in both upper and lower jaws and adjusting bridges. The patient asked me if I could do the work painlessly, knowing something of my work in pressure anesthesia, and having dreaded an operation he knew was inevitable if he wished to avoid a plate and get any use of his mal-occluding teeth.

I told him I believed I could do it, but as there were no cavities to work in with my cocain and pressure, I would have to make cavities, and that might be a little painful. I set to work and penetrated each tooth at the most available point, making a very small hole with a small bur. At the first show of sensitiveness I desisted, and filling the opening in both the tooth and my rubber tip with the cocain solution, I covered the opening and exerted considerable pressure at once. Now by holding long enough under hard and continuous pressure, one can almost invariably reach the pulp with the medicament in one effort, but I find it easier to relax and drill a little deeper (if I want to get ultimately into the pulp chamber) and then repeat the dose of cocain. I repeated in this case perhaps twice before reaching the pulp chamber, which I entered without pain. Then to make certain that my pulp was anesthetized to the apex, I prolonged my pressure treatment until I felt satisfied.

This was the procedure in each case of six teeth—incisors and bicuspids. I also cut off four elongated lower incisors and a cuspid by grinding, until I struck sensitive dentine, and then forcing cocain in, finished the grinding without pain. From one lower incisor I had to remove the pulp, exposed, without pain, by grinding. All the work described, the removal of pulps of six teeth and placing dressings, and grinding off the lower ones, was done in one and one-half hours. When ready to dismiss my patient I asked him to tell me how severely he had been hurt. He said that with the exception of the getting through the enamel, which was only suggestive of pain, the whole thing was no more painful than paring his nails. By any other process known to me I would have produced more pain than in the procedure, and would have had from four to six sittings of an hour or more each. The gentleman—a very busy man—appreciated the saving of time, as well as saving pain.

Now the method of performing this operation suggested to me a way to handle many cases that I believe we may be justified in doing. The rubber tips I have referred to are not available for all kinds and conditions of cavities, nor can they be made so and be effective. Again, many cavities are obscure and in position and condition to be very painful in opening them so as to begin to use the pressure method. I select another defective spot at a more available position on the tooth, as for instance, a little shallow cavity at the cervix, and utilize that. Many of these cervical cavities that are so sensitive may be thoroughly obtunded, even though the rubber tip covers but a portion of them, providing they are shallow enough to allow the tip to fit so as to confine the solution. The rubber readily fits quite a diversity of inequalities.

If I can not find a defect through which to make an opening, I feel justified in many cases, as I say, with the consent of my patient, to make an opening through sound tissue, selecting the place with judgment, and of course repairing the damage, as we would a decayed spot, by filling later. With such an opening you can so obtund the tooth as to be able to open into and excavate a cavity of decay without pain, bearing in mind always that you have no sensitiveness to warn you of trespassing upon dangerous ground.

Now I do not want to be understood as advising such a procedure as a common practice, nor in fact advising the pressure method in many cases where we can do without it. Each operator must decide for himself. If he wants to use the method I have devised, he may have an instrument at hand quite as convenient to pick up and go to work with as picking up a plugger, and one to be used at any stage of the cutting and preparing, if conditions will admit at all of its use. A little time spent in obtunding often saves time in the facility with which one can work afterward.

There are few medicaments that will infiltrate either dentine or pulp in any reasonable time, by simply lying in contact with it.—*March Review.*

